# Contribution of *excision repair cross-complementing group 1* genotypes to triple negative breast cancer risk

**DOI:** 10.1371/journal.pone.0202112

**Published:** 2018-08-10

**Authors:** Chia-Wen Tsai, Wen-Shin Chang, Te-Chun Shen, Chen-Hsien Su, Hwei-Chung Wang, Liang-Chih Liu, Da-Tian Bau

**Affiliations:** 1 Terry Fox Cancer Research Laboratory, Translational Medicine Research Center, China Medical University Hospital, Taichung, Taiwan; 2 Graduate Institute of Clinical Medical Science, China Medical University, Taichung, Taiwan; 3 Department of Bioinformatics and Medical Engineering, Asia University, Taichung, Taiwan; Duke Cancer Institute, UNITED STATES

## Abstract

Compared with other subgroups of breast cancer, triple negative breast cancer (TNBC) is considered to be the one with the greatest invasiveness and metastatic mobility, and the highest recurrence rate. Considering the lack of predictive markers for TNBC, we aimed to examine the contribution of excision repair cross complementing-group 1 (*ERCC1*) genotypes to TNBC. The rs11615 and rs3212986 of *ERCC1* were investigated and evaluated for their associations with susceptibility to breast cancer, especially TNBC, in Taiwan. In this study, 1,232 breast cancer patients (104 were TNBC) and 1,232 healthy controls were recruited and their genotypes at *ERCC1* rs11615 and rs3212986 were revealed by polymerase chain reaction restriction fragment length polymorphism (PCR-RFLP) analysis. Our results indicated that genotypes of *ERCC1* rs11615 (*P*_trend_ = 2.2*10E-9), but not rs3212986 (*P*_trend_ = 0.6181), were associated with breast cancer risk. In the allelic frequency distribution analysis, breast cancer patients carried the T allele of *ERCC1* rs11615 a higher rate than the control subjects, further supporting the idea that *ERCC1* rs11615 TT genotype is positively associated with breast cancer susceptibility. More importantly, the frequency of the *ERCC1* rs11615 TT genotype was even higher among TNBC patients than among other subtypes of breast cancer patients (*P* = 0.0001, odds ratio = 1.73, 95% confidence interval = 1.15–2.63). The genotypes of *ERCC1* rs11615 were not associated with Ki67 status. Our findings firstly show that the T allele of *ERCC1* rs11615 can serve as a predictive biomarker for breast cancer and TNBC. We believe that *ERCC1* could serve as a target for personalized treatment of breast cancer, especially for TNBC.

## Introduction

Published statistics reveal that breast cancer is the most common cancer diagnosed among females worldwide [1]. Among the subgroups of breast cancer, triple negative breast cancer (TNBC) accounts for 10–20% of all newly diagnosed female breast cancers [2]. Since cells of this cancer lack the three common receptors, estrogen receptor (ER), progesterone receptor (PR), and hormone epidermal growth factor receptor 2 (HER-2), there are as yet no specific clinical drugs or targeting therapies for this kind of breast cancer. As a result, TNBC is characterized by high invasiveness, poor prognosis, and high chances of recurrence [3]. The abnormality of gene expression in TNBC patients is another concern of scientists. Given the shortage of targeted treatments for TNBC and its typical properties, the discovery of the biomarkers and medication for TNBC are considered to be in a high priority.

Environmental factors, such as ionizing radiation (IR) and ultraviolet (UV) radiation, are considered to be common causes of DNA damage in living organisms [4]. In cases of DNA damage, repair systems such as base excision repair (BER), nucleotide excision repair (NER), and mismatch repair (MMR) are believed to confer resistance to front line cancer treatments [5]. Defective DNA repair systems enable cancer cells to accumulate genomic alterations, which may also cause them to lose their normal growth regulation [6–8]. Therefore, any polymorphisms in genes involved in DNA repair systems may contribute to the etiology of carcinogenesis, including breast cancer initiation and progression.

NER functions as the main pathway to repair massive DNA damage such as that caused by UV light, environmental mutagens, and some cancer chemotherapeutic adducts of DNA [9]. In literature, cellular NER capacity was found to be deficient in the cells of breast cancer patients, especially those at sporadic stage I [10, 11]. The deficiency could be explained by the lower expression of some NER proteins, such as XPA, XPF and CSB [11]. Among all the subtypes of breast cancer, the TNBC was of the lowest NER capacity [12]. The *excision repair cross complementing-group 1 (ERCC1)* gene is located on human chromosome 19q13.32, and encodes a DNA repair protein, ERCC1 [13]. ERCC1 plays an essential role in NER-pathway because of its damage recognition and excision ability [14]. A positive correlation was found between ERCC1 mRNA expression and DNA repair capacity in several studies [15, 16]. The expression of ERCC1 was increased by cisplatin treatment in a time- and a dose-dependent manner in ovarian cancer cell lines [17]. However, the expression level of ERCC1 may have a dual function in breast carcinogenesis. It was reported that higher expression of ERCC1 is associated with favorable prognostic factors for early stage breast cancer patients [18], but with poor outcome for those metastatic TNBC patients treated with platinum-based chemotherapy [19]. Mutations in this gene contributed to the etiology of cerebro-oculo-facio-skeletal (COFS) syndrome [20], and polymorphisms in *ERCC1* that alter its expression may influence overall genomic stability, and thus enhance personal susceptibility to cancer. Previously, *ERCC1* variants have been found to be associated with carcinogenesis in various types of cancer, such as lung, colorectal, gastric and ovarian, as well as breast cancer [21–25]. Notably, the association of *ERCC1* polymorphisms with breast cancer has been revealed in different countries, including Korea, United States, Iran, China and Thailand [20, 26–30]. As mentioned above, high expression of ERCC1 was found to be associated with poor patient outcomes for TNBC patients [18]. However, no previous literature has explored the contribution of *ERCC1* polymorphisms to TNBC.

In the current investigation, we aimed at discovering the contributions of *ERCC1* rs11615 and rs3212986 genotypes to breast cancer susceptibility in a large population of Taiwanese females, including 1,232 breast cancer cases and 1,232 healthy controls. These two SNPs were selected because they were reported to be associated with the susceptibility and outcomes of different cancers [21–25]. In addition, a query of the ClinVar and dbSNP databases turned out that these two SNPs were linked to “drug responses”. Interestingly, Zhu and his colleagues showed that there were no statistical associations between *ERCC1* rs11615 and the risk of breast cancer [30]. Moreover, we attempted to find in *ERCC1* a useful biomarker for early prediction and detection of TNBC in the Taiwanese population.

## Materials and methods

### Investigated sample collection

A total of 1,232 female patients diagnosed with breast cancer were enrolled in China Medical University Hospital (CMUH), Taichung, Taiwan. At the same time, controls were recruited from the Health Examination Cohort of CMUH [31]. These individuals had received a health checkup with history taking, complete physical examination, serial laboratory testing, and necessary image study. We excluded those with primary malignancy, metastatic cancer from other or unknown origin, and any hereditary or genetic disease. All the participants were volunteered, following self-administered questionnaires and furnishing of peripheral blood samples, for our following experiments. The content of the questionnaire includes former historical questions and the habits of alcohol consumption and cigarette smoking. These factors were recorded and further summarized in Table 1. All the enrolled individuals gave informed consent. Our study was evaluated and approved by the Institutional Review Board of China Medical University Hospital (DMR-99-IRB-108).

**Table 1:** Demographics and life-style of the investigated breast cancer patients and the control healthy women.

Characteristic	Controls (n = 1,232)			Patients (n = 1,232)			*P*-value
	n	%	Mean (SD)	n	%	Mean (SD)	
Age (years)							
< 40	359	29.1%		362	29.4%		0.89 ^a^
40–55	558	45.3%		547	44.4%		
> 55	315	25.6%		323	26.2%		
Age at menarche (years)			12.4 (0.7)			12.1 (0.6)	0.79 ^b^
Age at first birth of child (years)			29.4 (1.2)			29.8 (1.4)	0.63 ^b^
Age at menopause (years)			48.8 (1.8)			49.3 (2.0)	0.59 ^b^
Site							
Unilateral				1198	97.2%		
Bilateral				34	2.8%		
Family History							
First degree (Mother, sister, and daughter)				55	4.5%		
Second degree				6	0.5%		
No history				1171	95%		
Habit							
Cigarette smokers	86	7.0%		170	13.8%		<0.0001^a^
Alcohol drinkers	91	7.4%		162	13.1%		<0.0001^a^

Statistical results based on

^a^ Chi-squared or

^b^ unpaired *Student’s t*-test.

### Genotyping conditions

Genomic DNA extraction from each investigated individual was prepared using the QIAamp Blood Mini Kit (Blossom, Taipei, Taiwan). The DNA obtained from peripheral blood leucocytes was stored at –80°C after extraction. Amplified DNA products were subjected to digestion by BsrDI and MboII restriction endonucleases, respectively, for 2 h at 37°C. Detailed information on primer sequences and enzymatic digestion conditions is summarized in Table 2. Polymerase chain reaction (PCR) was performed following the manufacturer’s instructions on a BioRad Mycycler (BioRad, Hercules, CA, USA) after the digestion. For each PCR procedure, the conditions were set at 94°C for 5 min initial cycle; 35 cycles of 94°C for 30 s, 55°C for 30 s and 72°C for 30 s; and a final extension step at 72°C for 10 min. After PCR amplification, the PCR products were separated by 3% agarose gel electrophoresis for approximately 40 min. Following digestion with BsrDI, PCR products of *ERCC1* rs11615 originating from the C allele were uncut (393-bp), whereas the T allele was cut into fragments of 228-bp and 165-bp. Upon digestion with MboII, PCR products of *ERCC1* rs3212986 originating from the G allele were uncut (367-bp), while the C allele was cut into fragments of 233-bp and 134-bp. All the genotypic processing was repeated blindly by two researchers, and all the genotyping results were 100% concordant.

**Table 2:** The summary of primer sequences, polymerase chain reaction-based sequence and polymerase chain reaction-restriction fragment length polymorphisms (PCR-RFLP) for rs11615 and rs3212986 polymorphic sites on the *Excision Repair Cross-complementing Group 1* gene.

Polymorphisms (locations)	Primer sequences *	Restriction enzyme	SNP sequence	DNA fragment size (bp)
rs11615	F:5′-TTAGGAGGAGAGAGAAGCTG-3′	BsrDI	C	393 bp
	R:5′-GGCTTCTCATAGAACAGTCC-3′		T	228 + 165 bp
rs3212986	F:5′-AGGCTGTTTGATGTCCTGCA-3′	MboII	G	367 bp
	R:5’-AGAGGAAGAAGCAGAGTCAG-3′		T	+ 134 bp

* F and R indicate forward and reverse primers, respectively.

### Statistical analyses

All statistical analyses were performed using Stata 14.0. Student’s t-test was applied to the comparison of ages between the breast cancer case and control groups. Pearson’s Chi-square test was applied for comparing the distribution of the *ERCC1* genotypes among the control and breast cancer groups. The association between *ERCC1* genotypes and breast cancer risk was estimated by odds ratios (ORs) and 95% confidence intervals (CIs). Data differences were recognized as significant when the statistical *p*-value was less than 0.05. We calculated the statistical power of our analysis. With a sample size of 1,232 breast cancer cases and 1,232 controls, and a minor allele frequency of ~30% for both SNPs in controls, we had 80% power to detect a minimum OR of 1.29 for overall breast cancer risk. For TNBC risk, with 104 cases and 1,232 controls, we had 80% power to detect a minimum OR of 1.78.

## Results

### Comparison of demographics and lifestyles between the breast cancer case and control groups

The characteristics of the investigated population were summarized and shown in Table 1. Characteristics such as age, age at menarche, and the age at the first birth were all well-matched between patients and controls (*p*>0.05). Lifestyle factors like cigarette smoking and alcohol use were also considered in our study. The results revealed that both cigarette smoking and alcohol consumption were significantly different between groups. The number of smokers and alcohol drinkers in the patient group was much greater than that in the control group. The results demonstrated that these lifestyle-related factors may put the breast cancer patients at risk (*p*<0.0001). Amplified DNA products from the samples were digested by specific enzymes. Details are shown in Table 2.

### Association of *ERCC1* genotypes and breast cancer risk

In our current study, two *ERCC1* polymorphisms (rs11615 C>T and rs3212986 T>G) were studied and compared between healthy controls and breast cancer patients. Table 3 analyzed and demonstrated the distribution of *ERCC1* genotypes of each polymorphism between groups. The genotypes of *ERCC1* rs3212986 were not significantly different among healthy controls and breast cancer patients (*P*_trend_ = 0.6181, Table 3). In contrast, the distribution of *ERCC1* rs11615 genotypes was found to be differently distributed among 1,232 controls and 1,232 breast cancer patients (*P*_trend_ = 2.2 × 10^−9^) (Table 3). However, detailed analysis of the results for *ERCC1* rs11615 showed that it was the homozygous TT genotype, but not the heterozygous CT genotype, that related to the increasing risk of breast cancer (OR = 2.08 and 1.06, 95% CI = 1.64–2.64 and 0.89–1.26, *P*-value = 0.0001 and 0.5205, respectively) (Table 3). Recessive and dominant models of *ERCC1* rs11615 were further compared in Table 3. Both models indicated a great positive association between the genotypes of *ERCC1* rs11615 and breast cancer risk (OR = 2.03 and 1.29, 95% CI = 1.62–2.54 and 1.10–1.51, *P* = 0.0001 and 0.0016, respectively).

**Table 3:** Distribution of *Excision Repair Cross-complementing Group 1 (ERCC1)* genotypes among the breast cancer and the control woman.

Genotype	Controls		Patients		OR (95% CI)	*P*-value^a^
	n	%	n	%		
rs11615						
CC	616	50.0%	538	43.7%	1.00 (Reference)	
CT	477	38.7%	441	35.8%	1.06 (0.89–1.26)	0.5205
TT	139	11.3%	253	20.5%	**2.08 (1.64**–**2.64)**	0.0001*
*P*_trend_						**2.2 × 10**^−9^*
Carrier comparison						
CC+CT	1093	88.1%	979	79.5%	1.00 (Reference)	
TT	139	11.9%	253	20.5%	**2.03 (1.62**–**2.54)**	0.0001*
CC	616	50.0%	538	43.7%	1.00 (Reference)	
CT+TT	616	50.0%	694	56.3%	**1.29 (1.10**–**1.51)**	0.0016*
rs3212986						
TT	599	48.6%	576	46.7%	1.00 (Reference)	
GT	471	38.2%	483	39.2%	1.07 (0.90–1.27)	0.4606
GG	162	13.2%	173	14.1%	1.11 (0.87–1.42)	0.3974
*P*_trend_						0.6181
Carrier comparison						
TT+GT	1070	86.8%	1059	85.9%	1.00 (Reference)	
GG	162	13.2%	173	14.1%	1.08 (0.86–1.36)	0.5179
TT	599	48.6%	576	46.7%	1.00 (Reference)	
GT+GG	633	51.4%	656	53.3%	1.08 (0.92–1.26)	0.3536

^a^ *p*-value based on Chi-squared test without Yates’ correlation.

* Statistically identified as significant.

### Association of *ERCC1* allelic subtypes and breast cancer risk

To extend our study, we also analyzed the allelic frequencies of *ERCC1* polymorphisms (rs11615 and rs3212986) among the investigated groups (1,232 controls and 1,232 breast cancer patients); the data are presented in Table 4. This was consistent with our findings that the distribution of *ERCC1* rs11615 allelic frequencies was significantly associated with increased breast cancer risk (*P* = 8.8 × 10^−9^), while *ERCC1* rs3212986 allelic frequencies were not found to be related to breast cancer risk (*P* = 0.3028). In the patient group, the frequency of the variant T allele was much higher than the wild-type C allele (38.4% and 30.6%, respectively).

**Table 4:** Distribution of *Excision Repair Cross-complementing Group 1 (ERCC1)* allelic frequencies among the breast cancer patients and control women.

Allele	Controls	%	Patients	%	*P*-value^a^
rs11615					
Allele C	1709	69.4%	1517	61.6%	**8.8 × 10**^−9^*
Allele T	755	30.6%	947	38.4%	
rs3212986					
Allele T	1669	67.7%	1635	66.4%	0.3028
Allele G	795	32.3%	829	33.6%	

^a^ *P*-value based on Chi-squared test without Yates’ correlation.

* Statistically identified as significant.

### Association of *ERCC1* rs11615 genotypes with breast cancer risk

A Chi-square test was performed to investigate the association of *ERCC1* rs11615 genotypes with breast cancer risk. In the clinicopathologic characteristics analysis, there were 657 patients available for triple-negative status and 615 patients available for Ki67 status. Surprisingly, Table 5 revealed that *ERCC1* rs11615 genotypes were differentially distributed among the breast cancer patients who showed positive triple-negative status (*P* = 0.0001). However, a more representative distribution of *ERCC1* rs11615 genotypes was observed for the other factor, Ki67 status (OR = 1.05 and 1.05).

**Table 5:** Association of *Excision Repair Cross-complementing Group 1 (ERCC1)* rs11615 genotypes with breast cancer risk stratified by clinicopathologic characteristics compared with non-cancer healthy controls.

Character	Genotype, number (%)^a^			OR (95% CI)^b^	*P*-value^c^
	CC	CT	TT		
Control	616 (50.0)	477 (38.7)	139 (11.3)	1 (Reference)	
Triple-negative status					
No	265 (47.9)	205 (37.1)	83 (15.0)	1.09 (0.89–1.33)	0.0877
Yes	38 (36.5)	40 (38.5)	26 (25.0)	**1.73 (1.15**–2.63)*	0.0001*
Ki67 status					
Negative	135 (48.7)	103 (37.2)	39 (14.1)	1.05 (0.81–1.37)	0.4251
Positive	165 (48.8)	123 (36.4)	50 (14.8)	1.05 (0.82–1.33)	0.2054

^a^ Triple-negative and Ki67 status databases were available for only 657 and 615 patients, respectively. All data are given as number of patients (%) unless otherwise noted.

^b^ OR, odds ratio; CI, confidence interval, variant CT + TT versus CC.

^c^ Based on Chi-squared test.

* Statistical significant.

To summarize, these findings indicate that *ERCC1* rs11615 (C>T) was associated with breast cancer risk. Therefore, *ERCC1* rs11615 genotypes may serve as predictive markers for the early detection of breast cancer patients. More importantly, the variant forms of *ERCC1* genotypes (CT and TT) contribute to an increased risk of developing TNBC.

## Discussion

TNBC occurs more commonly in younger females, especially those with *BRCA1* germline mutations [32, 33]. Compared to other hormone receptor positive breast tumors, TNBC tumors are typically more invasive and aggressive, with greater risk of early relapse, which clinically enhances the difficulties of curing TNBC [34, 35]. Given the suboptimal outcomes after chemotherapy, the search for quantifiable TNBC biomarkers for early prediction is urgently needed. To this end, we have previously validated several biomarkers for TNBC in a large Taiwanese population. In 2014, the *Cyclin D1 (CCND1)* A870G GG genotype was found to be infrequent in Taiwanese TNBC patients, which may contribute to distinguishing the TNBC patients from other breast cancer patients [36]. In 2015, we found *X-Ray Repair Cross Complementing 3 (XRCC3)* genotypes were associated with Taiwanese TNBC patients, suggesting that XRCC3 may be a potential predictive marker for TNBC [31]. Further, in 2016, the CC genotype of *tissue inhibitor of metalloproteinase-1 (TIMP-1)* rs4898 was also found to increase the risk for TNBC in Taiwan and may serve as a predictive marker for TNBC [37]. The details of intracellular signaling pathways, such as the cell cycle, extracellular matrix regulation and DNA repair, are worth of further investigations.

The DNA repair system plays an essential role in preventing DNA damage accumulation, maintaining genomic stability and serving as anticancer gatekeepers of the cells. Several lines of evidence indicate that tumor cells were found with more DNA repair protein-related mutations, leading to partial or loss of their related functions, which may serve as one of the reasons for progression in cancer initiation and development [38–40]. Among the types of DNA damage, the double-strand breaks (DSBs) may represent the most severe and irreversible damage to the whole genome in the case they are not reversed by the DNA repair system immediately and properly when they are formed. Cells that survive DSBs and do not undergo apoptosis are prone to becoming cancer cells. Hence, we examined the genotypes of several DNA DSB repair genes, such as *X-Ray Repair Cross Complementing 3 (XRCC3)*, *XRCC6*, *XRCC7*, which are involved in the DSB repair system. The associations of these DNA repair genes with multiple types of cancer and diseases, including nasopharyngeal carcinoma [41], lung cancer [42], leiomyoma [43], breast cancer [44], hepatocellular carcinoma [45], and especially TNBC [46], have been explored in the literature.

Besides the genes involved in the DSB repair system, we are also interested in examining the contribution of the central NER repair protein, ERCC1, to the etiology of TNBC. The genotypes of *ERCC1* have been shown to be associated with other types of cancer, including colorectal cancer [22], bladder cancer [47], esophageal cancer [48], but not breast cancer, not to mention TNBC. The accumulated case-control results in other types of cancer showed that the genotypes of *ERCC1* may also contribute to TNBC, but this has never been investigated. In 2012, Ozkan and his colleagues demonstrated that the expression of ERCC1 was significantly elevated in approximately two thirds of the TNBC patients. More valuably, it may serve as a predictor for the poor response to platinum-based chemotherapy [49]. In 2015, Dumont and his colleagues proposed that the genotypes of *ERCC1* rs11615 and *CYP1B1* rs1056836 can jointly predict the prognosis responses to neoadjuvant chemotherapy of breast cancer patients, especially ER positive ones [50]. However, the sample size was small, with only 118 women, and *ERCC1* rs11615 could not serve as a TNBC marker. In addition, the need to combine with *CYP1B1* rs1056836 may add information to early prediction, but suggests that *ERCC1* rs11615 may serve as only a low-penetrant marker, but not a high-penetrant one. From the 5-fluorouracil-, doxorubicin- and cyclophosphamide-induced DNA repair viewpoint, the genotypes of either *ERCC1* rs11615 or rs3212986 may cause the differential responses to these drugs in TNBC or other subtypes of breast cancer patients [51]. The clinical study contained 324 breast cancer patients, of which the number of TNBC patients was 33, so any genotype’s association to TNBC is not conclusive or representative. In 2017, the prospective role of ERCC1 to be a promising marker for Caucasians was validated by El Kashef and his team [18]. In this study, we aim to validate the contribution of *ERCC1* genotypes for TNBC patients who are Taiwanese, the oriental Han population with a different genetic background from Caucasians. To fulfill this aim, we collected a large sample of 1,232 breast cancer patients in Taiwan, which strongly increased the credibility and the importance of our findings. We found that the genotypes of *ERCC1* rs11615 were associated with breast cancer susceptibility, while rs3212986 polymorphism was not. In detail, T allele (or CT and TT genotypes) of *ERCC1* rs11615 is a novel biomarker for Taiwanese females (Table 3). In addition, the T allele of *ERCC1* rs11615 was common in patients with breast cancer (Table 4). In a most recently updated meta-analysis in 2018 including 4,547 subjects, Li et al. reported that *ERCC1* rs11615 genotypes were associated with the risk of breast cancer, especially in Asian populations [52]. Among the breast cancer patients, we investigated, 104 of them were confirmed to be TNBC patients since their tissues were negative for ER, PR, and HER2/neu expression. Our findings indicate that the representation of the *ERCC1* rs11615 TT genotype was increased by about 10% (from 15% to 25%) among the patients with TNBC, compared with other breast cancer patients (Table 5). Inconsistent with this result, a previous study [46] reported that the overexpressed Ki-67 was a potential indicator for TNBC, it seems that the expression of Ki-67 has no linkage with the *ERCC1* rs11615 genotype in determining the susceptibility of TNBC in this study (Table 5).

In conclusion, the present case-control study, with a very large sample, indicates that the T allele of *ERCC1* rs11615 may potentially serve as a powerful marker for the prediction of breast cancer, especially for TNBC. Furthermore, it is the very first time that *ERCC1* rs11615 polymorphism was found to be associated with the risk of TNBC. The identification of *ERCC1* genotypes among Taiwanese individuals without cancer and those who suffer from breast cancer may lead to a better understanding of the mechanisms behind breast cancer. The feasibility of the *ERCC1* gene being a therapeutic target in drug development and an alternative treatment for TNBC may be quite promising.
